# IIIM-941, a Stilbene Derivative Inhibits NLRP3 Inflammasome Activation by Inducing Autophagy

**DOI:** 10.3389/fphar.2021.695712

**Published:** 2021-06-25

**Authors:** Mehboob Ali, Mehak Gupta, Abubakar Wani, Ankita Sharma, Mohd Abdullaha, Dilpreet Kour, Sushil Choudhary, Sandip B. Bharate, Gurdarshan Singh, Ajay Kumar

**Affiliations:** ^1^ PK-PD-Toxicology Division, CSIR-Indian Institute of Integrative Medicine, Jammu, India; ^2^ Academy of Scientific and Innovative Research (AcSIR), Ghaziabad, India; ^3^ Department of Immunology, St Jude Children’s Hospital, Memphis, TN, United States; ^4^ Natural Products and Medicinal Chemistry Division, CSIR-Indian Institute of Integrative Medicine, Jammu, India

**Keywords:** NLRP3 inflammasome, autophagy, ASC oligomerization, inflammation, AMP kinase, CAMKK2, calcium/calmodulin-dependent kinase kinase 2

## Abstract

Aberrant activation of NLRP3 inflammasome has been implicated in several inflammatory diseases. Autophagy is one of the primary mechanisms that regulate NLRP3 inflammasome activity. In this study, we attempted to target NLRP3 inflammasome activity by a synthetic compound IIIM-941. We found that IIIM-941 inhibits ATP induced NLRP3 inflammasome by induction of autophagy through AMPK pathway in bone marrow derived macrophages (BMDMs) and J774A.1 cells. It was interesting to observe that IIIM-941 did not show any inhibitory activity against LPS induced pro-inflammatory cytokines TNF-α and IL-6. The anti-NLRP3 activity of IIIM-941 was significantly reversed when we attempted to block autophagy by using either pharmacological inhibitor bafilomycin A1or by using siRNA against AMPK. Further, we found that IIIM-941 downregulated the expression of NLRP3 and prevented the oligomerization of ASC to exert its anti-NLRP3 inflammasome effect in J774A.1 cells. We validated inhibitory activity of IIIM-941 against NLRP3 in three different mice models. The anti-inflammatory effect of IIIM-941 was highly significant in ATP induced peritoneal inflammation model. IIIM-941 was similarly effective in suppressing MSU induced IL-1β in the air pouch model of inflammation without affecting the levels of TNF-α and IL-6. Finally, oral efficacy of IIIM-941 was also proved in MSU indued foot paw edema model of inflammation in mice at 10 and 20 mg/kg (b.w.). The compounds like IIIM-941 can be explored further for the development of therapies against diseases such as Alzheimer’s disease and Parkinson’s disease, where hampered autophagy and NLRP3 activation play a crucial role in the pathological development.

## Introduction

NLRP3 inflammasome is a part of inflammatory innate immune response of the host against invading pathogens and endogenously generated damage signals. The activation of NLRP3 inflammasome is involved in the maturation and secretion of IL-1β and IL-18 to their active forms. These cytokines act as a defense mechanism to protect the cell from potentially harmful signals (Latz et al., 2013; Man and Kanneganti, 2016). However, under certain circumstances, chronic stimulation of NLRP3 inflammasome can lead to development of inflammatory diseases (Ozaki et al., 2015; Wang et al., 2017). Apart from infectious diseases, several chronic inflammatory conditions such as Alzheimer’s disease, cryopyrin-associated periodic syndrome (CAPS), gouty arthritis, diabetes mellites, Parkinson’s disease, multiple sclerosis etc. have been linked with the aberrant activation of NLRP3 inflammasome (Zhang et al., 2021). The involvement of NLRP3 inflammasome in multiple diseases makes it an important target to develop new drugs against these diseases. In this context, autophagy has emerged as one of the key processes that regulate inflammasomes and related inflammation. Autophagy is a natural homeostatic process, which involves the degradation and recycling of cellular components by delivering them to lysosomes (Behrends et al., 2010; Biasizzo and Kopitar-Jerala, 2020). Autophagy plays an important role in cell protection and helps in degradation of pathogens invading the cell such as intracellular protozoa, bacteria, and viruses (Deretic, 2005; Levine and Deretic, 2007; Schmid and Munz, 2007).

In one of the earliest reports linking autophagy and inflammasome, increased activity of caspase-1 and cleavage of IL-1β was observed in mouse fetal liver-derived macrophages when an autophagy-related protein ATG16L1 was knocked out (Saitoh et al., 2008). More recent studies have shown that autophagy can control the aberrant activation of inflammasomes at multiple levels including autophagic clearance of components of inflammasomes, cytokines or inflammasome activator endogenous damaging signals (Harris et al., 2017). Autophagy helps in the destruction of worn-out organelles such as mitochondria, thus, it helps in downregulating the activity of NLRP3 inflammasome by removal of mtDNA and mtROS (Rubartelli, 2012; Navarro-Yepes et al., 2014; Qiao et al., 2016). Additionally, the knockdown of autophagy related genes Atg5 or Atg7 in macrophages is reported to increase the levels of IL-1β in inflammatory conditions like uveitis, liver fibrosis, and colitis (Lodder et al., 2015; Lee et al., 2016; Santeford et al., 2016)). The accruing evidence suggests that autophagy may be a primary mechanism to control excessive inflammatory response against different PAMPs and DAMPs. The increased activity of inflammasome prompts the activation of autophagy, which ultimately leads to downregulation of inflammasome activity. However, under pathological conditions, the autophagy may not be able to properly regulate the inflammasome activity. Therefore, pharmacological inventions can be developed to activate autophagy for inhibition of NLRP3 inflammasome.

In this study, we analyzed in detail the anti-NLRP3 activity of compound IIIM-941 (mono-chloro tri-methoxy substituted synthetic stilbene). This compound was identified as a potential inhibitor of NLRP3 inflammasome in our screening of a series of compounds synthesized from an ERβ (estrogen receptor β) agonist IIIM-983 (compound 11), which was also reported to inhibit IL-1β (Mewshaw et al., 2005). We show here that IIIM-941 inhibits ATP induced NLRP3 inflammasome in the in BMDMs and J774A.1 cells. Our data indicate that IIIM-941 exhibits anti-NLRP3 inflammasome activity by inducing autophagy. In this study, we have also validated the anti-NLRP3 effect of IIIM-941 in three different mice models.

## Materials and Methods

### Reagents and Antibodies

*Chemicals:* Dulbecco’s modified eagle medium (DMEM) (D1152), streptomycin (S6501), penicillin (P3032), triton X-100 (T8787), bafilomycin A1 (B1793), sodium bicarbonate (S5761), phosphate buffer saline (PBS) (D5652), 4, 6-diamidino-2-phenylindole (DAPI) (D9542), dimethylsulfoxide (D2650), N,N′-methylene bis acrylamide (M7279), sodium orthovanadate (450,243), sodium fluoride (215,309), tween 20 (P7949), ammonium persulfate (A3678), TEMED (T7024), glycerol (G5516), HEPES (H3375), paraformaldehyde (P6148), protease inhibitor cocktail (P8340), MTT (M5655), trizma (T6066), uric acid sodium salt (U2875), SDS (L3771), suberic acid bis(N-hydroxysuccinimide ester) (S1885), lipopolysaccharide (LPS; L3129), and ATP (A6419) were bought from Sigma-Aldrich. acrylamide (193,982), glycine (194,825), albumin bovine fraction V (160,069), and phenylmethylsulfonyl fluoride (PMSF; 195,381), were purchased from MP biomedical. sodium chloride (MB023) and skimmed milk (GRM1254) were bought from Himedia. OPTI-MEM media (Gibco, 11,058–021), strataclean resin (Agilent, 400,714–61). EDTA (15,575–038), ELISA kits for TNFα, IL-1β and IL-6 were purchased from invitrogen (eBioscience).

*Antibodies and siRNA:* BECN1 (SC-48341), ATG5 (SC-133158), ASC (SC-22514)), CASP1 (SC-56036), mouse scrambled siRNA (Sc-37007), and HRP-linked anti-goat IgG (SC-2354), were bought from Santa Cruz Biotechnology. AMPK (2532S), pAMPK(2535S), cleaved CASP1 (ASP296) (89332S), NLRP3 (15101S), siRNA AMPK (6620S), pMTOR Ser 2448 (5536S), MTOR (2972S), pCaMKK2 Ser 511(12818S), anti-mouse IgG Alexa flour 488 (4412S), anti-rabbit IgG Alexa flour 555 (4409S), HRP-linked rabbit IgG (7074S), and HRP-linked mouse IgG (7076S) were purchased from cell signaling and technology. M-CSF (416-ML/CF), anti-IL-1β (AF-401-NA) were purchased from R and D biotechnology. Anti-LC3B-II (L7543), pLKB1 Ser 428 (SAB4504034) and anti-ACTB (A3854) were purchased from Sigma-Aldrich.

PVDF Membrane (ISEQ00010), ECL-kit (WBKLS0500) from Millipore, FuGENE HD (E2313) from Promega, Precision plus protein markers (161–0,375) and Bradford reagent (500–0,006) were purchased from Bio-Rad.

### Cell Culture

Mouse macrophages J774A.1 were cultured in DMEM media supplemented with 10% fetal bovine serum (FBS), streptomycin (100 mg/L) and penicillin G (70 mg/L). Cells were incubated at 37°C with 95% humidity and 5% CO_2_ in the CO_2_ incubator.

### Bone Marrow-Derived Macrophages

Bone marrow was derived from femur and tibia of Balb/C mice and the cells were differentiated for 7 days in DMEM media with M-CSF (20 ng/ml) or in DMEM with 10% heat-inactivated FBS and 30% L929 conditional media.

### NLRP3 Inflammasome Activity in J774A.1 Cells and Bone Marrow-Derived Macrophages

In six well and 24 well plates, J774A.1 cells seeded at a density of 1 × 10^6^ and 0.4 × 10^6^ cells per well respectively. The cells were primed for 5.5 h with LPS (1 μg/ml) and then media was replaced with serum-free media containing either DMSO, MCC950 (100 nM), or IIIM-941 for 1 h. The inflammasome activator ATP (3 mM) was added for 30 min in order to stimulate the cells.

BMDMs were primed with LPS (20 ng/ml) for 4 h followed by treatment with either DMSO, MCC950, or IIIM-941 for 1 h in serum-free media and then stimulated with ATP (3 mM) for 45 min before analysis through ELISA for release of IL-1β.

### Western Blotting

After the completion of treatments, cell lysates were prepared in the RIPA buffer and 60 µg of sample/protein was loaded for SDS-PAGE. For western blotting of proteins released in the media, Strataclean resin was used to concentrate the proteins from the supernatant samples by using protocol provided by the manufacturer. The proteins were resolved on SDS-PAGE and transfered to PVDF membrane under cold condition. The blocking of blots was done using 5% BSA or skimmed milk at RT for 1 h and then incubated overnight at 4°C with primary antibody of interest. The protein blots were then incubated with HRP-conjugated secondary antibody for 1.5 h at RT. Millipore Immobilon western chemiluminescent HRP substrate was used to develop the blots, and the signal was captured on X-ray film or Chemidoc system (Syngene G:BOX Chemi XT 4).

### ASC Oligomerization

After the completion of treatments, the cells were lyzed in the ice-cold buffer (KCl (150 mM), NP-40 (1%), HEPES-KOH (20 mM, pH 7.5), PMSF (0.1 mM), sodium orthovanadate (1 mM), protease inhibitor cocktail (1%). Cell lysates were centrifuged at 330 g for 10 min 50 µL of supernatants were collected for western blotting and pellets were washed and resuspended in 500 µL cold PBS. For the crosslinking of ASC, 2 mM of suberic acid was added to the resuspended pellet and incubated at 37°C for 30 min. The centrifugation was done to collect the crosslinked pellet at 330 g for 10 min at 4°C. The crosslinked pellet was dissolved in 30 µL of 2x Laemmlli dye. The samples were heated at 95°C for 5 min and analyzed by western blotting.

### Lactate Dehydrogenase Activity Assay

Lactate dehydrogenase activity (LDH) was analyzed from the supernatants by lactate dehydrogenase assay kit as per the manufacture’s instructions (Sigma-Aldrich).

### Enzyme-Linked Immunosorbent Assay

The level of cytokines was analyzed by the ELISA assay as per the manufacture’s instruction (Invitrogen or eBiosciences). After various treatments including NLRP3 inflammasome activation, the supernatants were collected to analyze the level of cytokines (IL-1β, TNFα, and IL-6) as reported earlier (Gupta et al., 2018). The cytokines levels were normalized by dividing the values with total protein content of each sample.

### Cell Viability Assay

The cell viability was determined by MTT assay. J774A.1 cells or BMDMs were seeded at a density of 0.4 × 10^6^ per well of 96 well plates. Cells were treated with different concentration of IIIM-941 for 24 h, MTT was added at a concentration of 2.5 mg/ml for 4 h before the termination of treatments. The supernatants were discarded and replaced with DMSO and absorbance was measured at 570 nm.

### Confocal Microscopy

J774A.1 cells were seeded at a density of 1 × 10^6^ cells per well in six well plates containing coverslips. After various treatments, the cells were given PBS wash and cell fixation was done using 4% paraformaldehyde for 30 min at room temperature. Triton X-100 (0.2%) was added for 7 min to permeabilize the cell and then blocked with buffer containing 2% BSA and 0.2% triton-100 in PBS for 1 h. The cells were further incubated with the primary antibody overnight under cold condtions followed by the incubation with secondary antibody (Alexa flour 488 or Alexa flour 555) next day at room temperature for 1 h. The cells were washed in PBS and further incubated with DAPI. The mounting media (glycerol and PBS) was added before taking images at either Olympus FV-1000 confocal laser scanning microscope or Yokogawa CQ1 Benchtop High-Content Analysis System at 60x.

### Transfection of J774A.1 Cells With siAMPK

Transfection was done in J774A.1 cells in six well plates wherein the cells were seeded at a density of 0.7 × 10^6^ per well. The cells were incubated in OPTI-MEM media for 30 min before adding AMPK siRNA in FuGENE HD for 24 h. After the transfection, the cells were primed with LPS (1 μg/ml) followed by stimulation with ATP (3 mM). The treatment with IIIM-941 was given 1 h before ATP and the samples were further processed for western blotting.

### Adenosine 5′-Triphosphate Levels

The concentration of ATP was measured in J774A.1 cells to analyze the time-dependent effect of IIIM-941 and secondly to check the concentration-dependent effect of IIIM-941 under the NLRP3 inflammasome activation conditions on ATP. Briefly, after various treatments, the cell lysates were prepared by using RIPA buffer under cold conditions and the lysates were used to analyze the concentration of ATP by using ATP Bioluminescent Assay Kit (FLAA, Sigma-Aldrich) as per the manufacture’s instructions. This assay is based on the fact that when luciferase catalyzes the oxidation of D-luciferin, ATP is consumed, and the light is released. The ATP content was evaluated using a luminometer based on amount of the light emitted. The cellular ATP content was normalized by total protein contained in the sample.

### Animals and Ethics Statement

Balb/C female mice weighing 20–25 g were used in the study with the proper acclimatization in the housing conditions at 25°C on 12 h dark and light cycles for 5–7 days. The animals were sacrificed by CO_2_ inhalation in euthanasia chamber at the end of the experiments. All the animal studies were done in compliance with the guidelines of institutional animals ethics committee (IAEC), IIIM, Jammu, India. The protocols for all the animal experiments were approved by IAEC (IAEC/1894/76/2/20, IAEC/1899/77/8/20 and IAEC/1924/77/8/20).

### Drug Formulation

For *in vivo* studies, IIIM-941 was formulated in 5% of DMSO, 30% PEG400, 20% PEG200 and 45% distilled water. LPS and ATP were dissolved in PBS.

### Peritoneal Inflammation

Female Balb/C mice were divided into different groups. The groups were 1) control, 2) vehicle 3) LPS, 4) LPS + ATP, 5) IIIM-941 5 mg/kg + LPS + ATP 6) IIM-941 10 mg/kg + LPS + ATP and 7) IIIM-941, 20 mg/kg + LPS + ATP) with five animals in each group (*n* = 5). The mice were intraperitoneal injected with IIIM-941 (20, 10 and 5 mg/kg) 30 min prior to the administration of LPS (2 mg/kg) for 4 h. ATP was administrated intraperitoneally 100 mM for 15 min. Then 3 ml of incomplete DMEM was injected and the peritoneal lavage was collected. The level of IL-β, IL-6 and TNFα were analyzed by ELISA from the peritoneal lavage.

### Air Pouch Model

We used air pouch model to observe inflammation induced by MSU crystal in Balb/C mice. Balb/C female mice were divided into six groups with five (*n* = 5) animals in each group. Animals were grouped as 1) control 2) vehicle 3) colchicine (1 mg/kg) and MSU 4) IIIM-941 (10 mg/kg) with MSU 5) IIIM-941 (20 mg/kg with MSU and 6) MSU only. Air pouch was developed by injecting 4 ml of sterile air in the dorsum of the Balb/c female mice. On the third day the dorsum was again raised to maintain the pouch size. On the sixth day Monosodium urate (MSU) salt (3 mg/ml) was injected in the pouches. The treatments with IIIM-941 (10 and 20 mg/kg) and colchicine (1 mg/kg) were given 30 min before the injection of MSU crystal for 6 h. Mice were sacrificed and the lavages were collected from air pouches and the level of IL-1β was measurd by ELISA and western blot, whereas levels of IL-6, and TNF-α were analyzed by ELISA.

### MSU Foot Pad Paw Model

Balb/C female mice were used in this study and were grouped into six groups with five animals in each group (*n* = 5). Animals were grouped as 1) control 2) vehicle 3) MSU 4) IIIM-941 (10 mg/kg) + MSU 5) IIIM-941 (20 mg/kg) + MSU 6) colchicine (1 mg/kg) + MSU. Mice were injected subcutaneously with monosodium urate (5 µg/mice) in the right foot pad for 24 h. IIIM-941 (10 and 20 mg/kg) and colchicine treatments were given orally 30 min prior to the MSU injection. The tissues were homogenized in RIPA buffer after being isolated from the foot paw. The lysates were analyzed for levels of different proteins through western blotting.

### Statistical Analysis

The values presented here are the mean ± SD. Statistical comparison involving multiple samples or groups was done by using one way ANOVA. Bonferroni test was used as a post hoc to calculate statistical significance, *p* value < 0.05 was considered significant, ***p < 0.001, **p < 0.01, *p < 0.05. Instat-3 and Graphpad Prism V softwares were used to anlayze the data.

## Result

### IIIM-941 Inhibited IL-1β Activation Without Affecting Other Inflammatory Cytokines and Inducing Cytotoxicity

**FIGURE 1:**
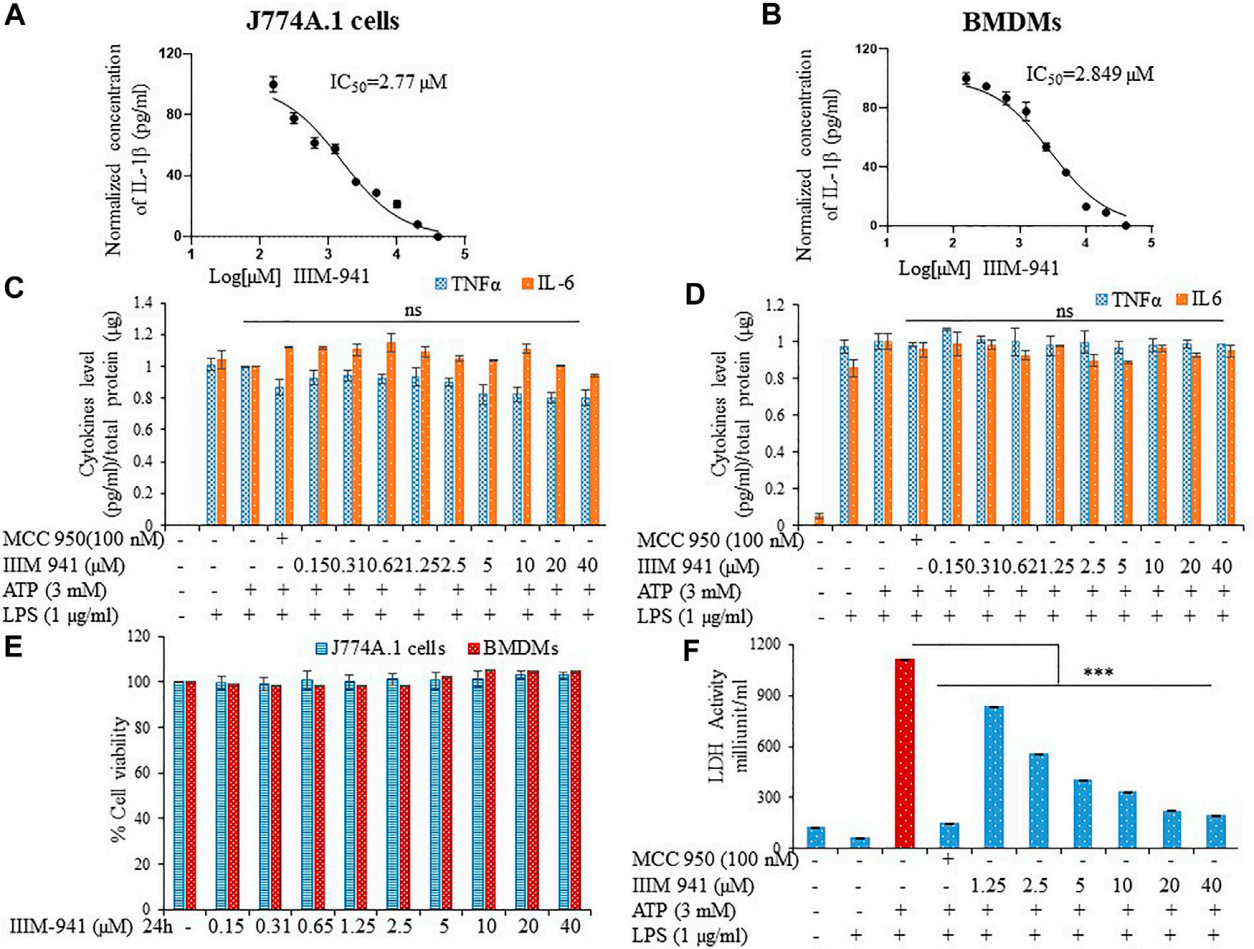
IIIM-941 inbihited NLRP3 inflammasome activation without affecting TNF-α and IL-6 in J774A.1 cells and BMDMs. J774A.1 cells and BMDMs were primed with LPS (1 µg) for 5.5 h and then different concentrations of IIIM-941 and MCC950 (100 nM) were added for 1 h before ATP (3 mM) stimulation for 30 min **(A) (B)** IC50 of IIIM-941 in J774A.1 and BMDMs **(C) (D)** IL-6 and TNF-α levels were analyzed in the supernatants of treated J774A.1 and BMDMs cells by ELISA **(E)** MTT assay to determine cell viability in J774A.1 and BMDM cells treated with different concentration of IIIM-941 for 24 h. Data are presented here as % cell viability where control samples are considered as fully viable **(F)** LDH released in the supernatants was measured by LDH activity assay kit. Data presented here are of three independent experiments ±SD. Statistical comparisons were done between LPS + ATP, MCC950 and IIIM-941 treated samples as mentioned in the materials and methods. *p* values ***p < 0.001, **p < 0.01, *p < 0.05.

We screened several analogs of ER-β agonist IIIM-983 for anti- NLRP3 inflammasome activity. We identified IIIM-941 as one of the potential inhibitors of NLRP3 inflammasome. We found that IIIM-983 inhibited NLRP3 inflammasome only when added at the first step that is before priming by LPS, whereas IIIM-941 displayed NLRP3 inflammasome inhibition at both the steps that is LPS priming and before stimulation by ATP in J774A.1 macrophages (Supplementary Figures S1B,C). The complete screening data have already been published (Abdullaha et al., 2021). In this study, we attempted to further explore in detail, the anti-NLRP3 inflammasome activity of IIIM-941. We calculated the anti-NLRP3 inflammasome IC50 value of IIIM-941 in two cell types that is J774A.1 and primary bone marrow derived macrophages (BMDMs) in presence of ATP as a second stimuli. The IC50 values in J774A.1 and BMDMs came out to be 2.77 and 2.84 µM respectively with IL-1β release as a read out for the activity of NLRP3 inflammasome (Figures 1A,B and Supplementary Figures S1D,E). We further analyzed whether IIIM-941 can have similar effect on other proinflammatory cytokines TNFα and IL-6 in both types of cells. The results clearly indicated that IIIM-941 just like MCC950 inhibits activation of IL-1β without affecting TNFα and IL-6 (Figures 1C,D). The anti-inflammatory activities compounds are sometimes caused by their cytotoxic and overall suppressive effect on the functioning of cells. Therefore, we assessed if IIIM-941 is toxic to the cells by treating the both types of cells in concentration dependent manner. Interestingly, IIIM-941 did not show any toxicity after a treatment of either J774A.1 or BMDMs even at a concentration of 40 µM (Figure 1E). Further, the anti-NLRP3 inflammasome activity of IIIM-941 was evident in the LDH release assay, where, LPS + ATP induced LDH release was significantly inhibited by IIIM-941 in a concentration dependent manner (Figure 1F).

### IIIM-941 Inhibited the Release of Cleaved IL-1β and Caspase-1 by Preventing the Assembly of NLRP3 Complex

**FIGURE 2:**
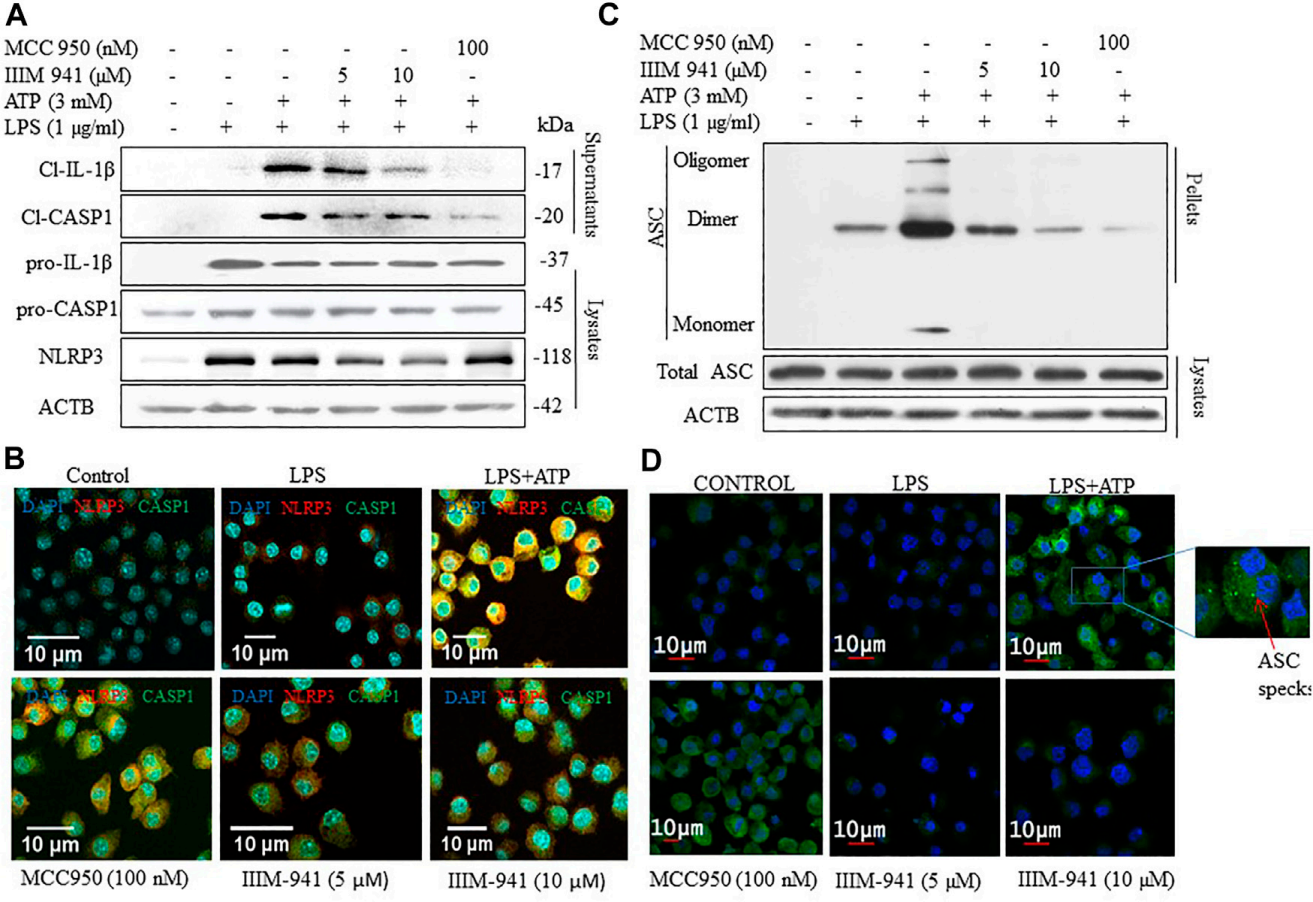
Effect of IIIM-941 on proteins involved in NLRP3 inflmmasome complex formation. J774A.1 cells were treated similarly as in Figure 1 before anlaysis for **(A)** Cleaved CASP1 and cleaved IL-1β in the supernatant and pro-CASP1, pro-IL-1β, NLRP3 in the cell lysates through western blotting **(B)** Colocalization of NLRP3 and CASP1 in J774A.1 cells thorough confocal microscopy. Red and green fluorescence represents NLRP3 and CASP1 respectively, and nucleus is stained blue with DAPI. Scale bar for the confocal images was drawn by using ImageJ software. Fluorescence quanitification of these images is given in the Supplementary Figure S2B **(C)** ASC oligomerization in cross-linked cytosolic pellets and ASC expression in cell lysate in J774A.1 cells by western blotting **(D)** ASC speck formation determined by confocal microscopy in J774A.1 cells. Green dots or specks represent ASC oligomerization. ImageJ software was used to quantify the band density of the western blots displayed in figure A and C, the quantification data are presented in Supplementary Figures S2A,C.

After confirming the anti-NLRP3 inflammasome activity of IIIM-941, we further wanted to analyze the effect of IIIM-941 on the proteins involved in NLRP3 inflammasome formation. Therefore, we primed the J774A.1 cells with LPS and treated them with IIIM-941 for 1 h before stimulation with ATP. We found that IIIM-941 at 5 and 10 µM inhibited the release of cleaved fragments of CASP1 and IL-1β in the media when compared to LPS + ATP treated samples (Figure 2A and Supplementary Figure S2A). Whereas, the pro-caspase-1 and pro-IL-1β levels remained unchanged after treatment with IIIM-941 (Figure 2A). Interestingly, IIIM-941 significantly inhibited the expression of NLRP3 under similar treatment conditions (Figure 2A and Supplementary Figure S2A).

We confirmed the downregulatory effect of IIIM-941 on the expression of NLRP3 and CASP1 through confocal microscopy. Wherein under similar conditions as in Figure 2A, there was significant reduction in the expression of NLRP3 and CASP1, which was visible in the form of reduced red and green fluorescence in the cells treated with IIIM-941 at 5 and 10 µM (Figure 2B and Supplementary Figure S2B). The results also confirmed the reduced co-localization of NLRP3 and CASP1.

We further wanted to know if IIIM-941 affects oligomerization of ASC protein. Therefore, under similar conditions, we treated the J774A.1 cells with IIIM-941 and we found that IIIM-941 inhibits the oligomerization of ASC, which was confirmed by western blotting, where cells treated with 10 µM of IIIM-941 displayed almost complete inhibition of ASC oligomerization (Figure 2C and Supplementary Figure S2C). However, total ASC levels were not changed under similar experimental conditions (Figure 2C). Inhibition of ASC oligomerization was also confirmed by using confocal microscopy, where ASC speck formation (indicating ASC oligomerization) was significantly reduced from 40% in LPS + ATP activated cells to 18% in IIIM-941 treated cells (Figure 2D and Supplementary Figure S2D).

### IIIM-941 Induced Autophagy in J774A.1 Cells by AMPK Pathway in Presence or Absence of NLRP3 Inflammasome Activation Conditions

**FIGURE 3:**
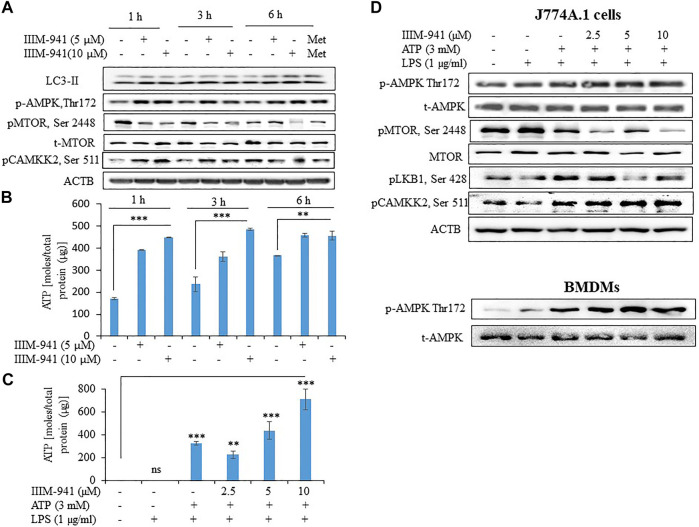
IIIM-941 activated AMPK pathway through pCAMKK2 upregulation **(A)** Western blots analysis of various proteins to ascertain the time-dependent effect of IIIM-941on autophagy and AMPK pathway in J774A.1 cells. Metformin (100 μM) treatment for 6 h was used as a standard in this experiment **(B)** Time-dependent effect of IIIM-941 on cellular ATP as measured by amount of bioluminescence produced due to luciferase activity. The samples were normalized by dividing the total quantity of ATP by total protein present in the sample **(C)** Effect of IIIM-941 on cellular ATP after treatment of cells under NLRP3 inflammasome activation conditions **(D)** Western blots showing the effect of IIIM-941 on AMPK pathway under NLRP3 inflammasome activation conditions. Data presented in Figure B and C are average ±SD of three independent experiments. *p* value < 0.05 was considered significant, ***p < 0.001, **p < 0.01, *p < 0.05.

Induction of autophagy negatively regulates the activation NLRP3 inflammasome. In order to determine whether autophagy plays any role in the IIIM-941 mediated inhibition of NLRP3 inflammasome, we treated the J774A.1 cells for different time periods and analyzed its effect on conversion of LC3-I to LC3-II. The cells treated with IIIM-941 displayed significant conversion of LC3-I to LC3-II at all the time points that is 1, 3 and 6 h (Figure 3A). Additionally, the increased expression of pAMPK (Thr-172) indicated the involvement of AMPK pathway in autophagy induced by IIIM-941 (Figure 3A). We further observed significant inhibition of pMTOR (Ser 2448), which is a downstream target in the AMPK pathway (Figure 3A). AMPK activation may directly be regulated by the levels of ATP present in the cell. Therefore, we checked the time-dependent effect of IIIM-941 on the intracellular levels of ATP. Our data clearly indicated that IIIM-941 do not negatively affect ATP concentration in the cells, on the contrary, significant rise in ATP levels was observed (Figure 3B). We again checked the concentration-dependent effect of IIIM-941 on the ATP under NLRP3 inflammasome activation conditions. The results showed almost similar pattern as observed under time-dependent conditions (Figure 3C). These data indicated the involvement calcium/calmodulin pathway through calmodulin-dependent protein kinase 2 (CAMKK2) for the activation of AMPK pathway. Our data confirmed this assumption, when IIIM-941 treatment led to increased expression of pCAMKK2 (Ser 511) in J774A.1 cells when treated for 1 and 3 h respectively, however, a decreased expression of pCAMKK2 (Ser 511) was observed after 6 h of treatment (Figure 3A).

To confirm these findings under the conditions used for NLRP3 inflammasome activation, we treated the J774A.1 cells and BMDMs with IIIM-941. We found that 1 h treatment of IIIM-941 at different concentrations (2.5, 5.0 and 10 µM) caused the phosphorylation and activation of AMPK at Thr-172 (Figure 3D and Supplementary Figure S3B). We further observed that IIIM-941 could also inhibit pMTOR (Ser 2448) and pLKB1 (Ser 428) under these conditions in J774A.1 cells (Figure 3D and Supplementary Figure S3B). It was further interesting to note that IIIM-941 caused significant activation of CAMKK2 (Ser 511) under NLRP3 inflammasome activation conditions as well (Figure 3D and Supplementary Figure S3B).

### IIIM-941 Induced Autophagy to Suppress NLRP3 Inflammasome Activation in J774A.1 and BMDM Cells

**FIGURE 4:**
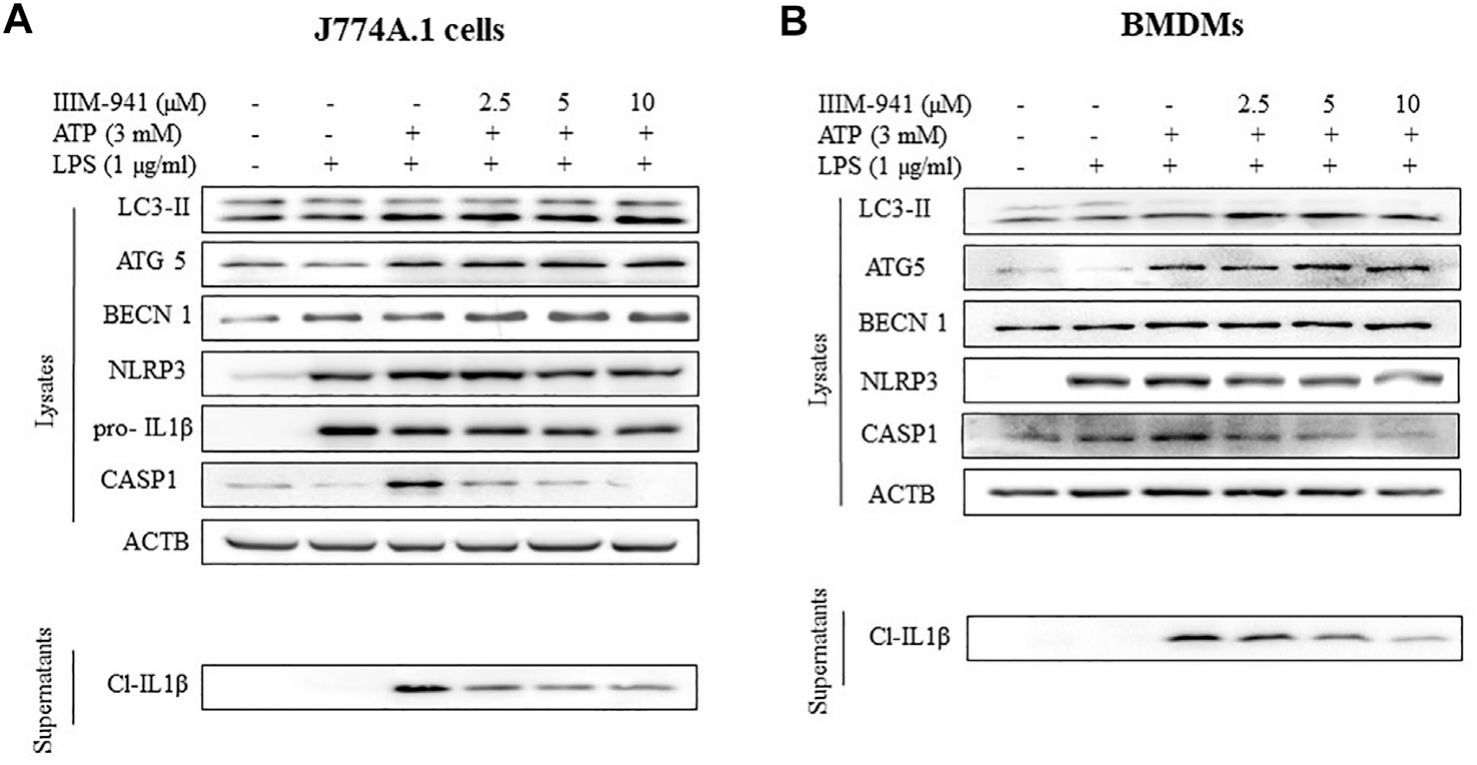
IIIM-941 inhibited NLRP3 inflammasome by inducing autophagy in J774A.1 and BMDM cells **(A)** IIIM-941 at 5 and 10 µM increased the expression of pAMPK (Thr-172) and induced autophagy as represented by increased levels of LC3-II in a time dependent manner in J774A.1 cells as analyzed by western blotting **(B)** Western blotting anlayis of autophagy and NLRP3 inflammasome proteins was done under the conditions used for induction of NLRP3 inflammasome in J774A.1 cells and **(C)** in BMDMs. For induction of NLRP3 inflammasome, similar conditions were used as in Figure 1A. The blots presented here are representative in nature. ImageJ software was used to quantify the band density of the western blots. The quantified data of three independent experiments ±SD for Figure **B** and **C** is presented in the Supplementary Figures S3A,B. The quantified data presented here for Fig A is from three independent experiments ±SD. *p* value < 0.05 was considered significant, ***p < 0.001, **p < 0.01, *p < 0.05.

Further, we wanted to know if IIIM-941 could induce autophagy under the conditions used for NLRP3 inhibition assay and whether the induction of autophagy is correlated with levels of inflammasome proteins. Interestingly, IIIM-941 triggered the conversion of LC3-I to LC3-II in J774A.1 cells primed with LPS and stimulated with ATP, thus indicating the induction of autophagy (Figure 4B and Supplementary Figure S4A). Besides, IIIM-941 also induced the expression of other important proteins ATG5 and BECN1 (beclin-1) involved in autophagy (Figure 4B and Supplementary Figure S4A). However, as observed in Figure 2A, the effect of IIIM-941 on NLRP3 inflammasome proteins remained same (Figure 4B and Supplementary Figure S4A). We also attempted to analyze the effect of IIIM-941 on autophagy induction in BMDMs under similar conditions. Interestingly, IIIM-941 affected the autophagy proteins LC3-II, ATG5, BECN1 and NLRP3 inflammasome proteins in a similar way as observed in J774A.1 cells (Figure 4C and Supplementary Figure S4B).

### Inhibition of Autophagy Led to the Reversal of the Inhibitory Effect of IIIM-941 on NLRP3 Inflammasome

**FIGURE 5:**
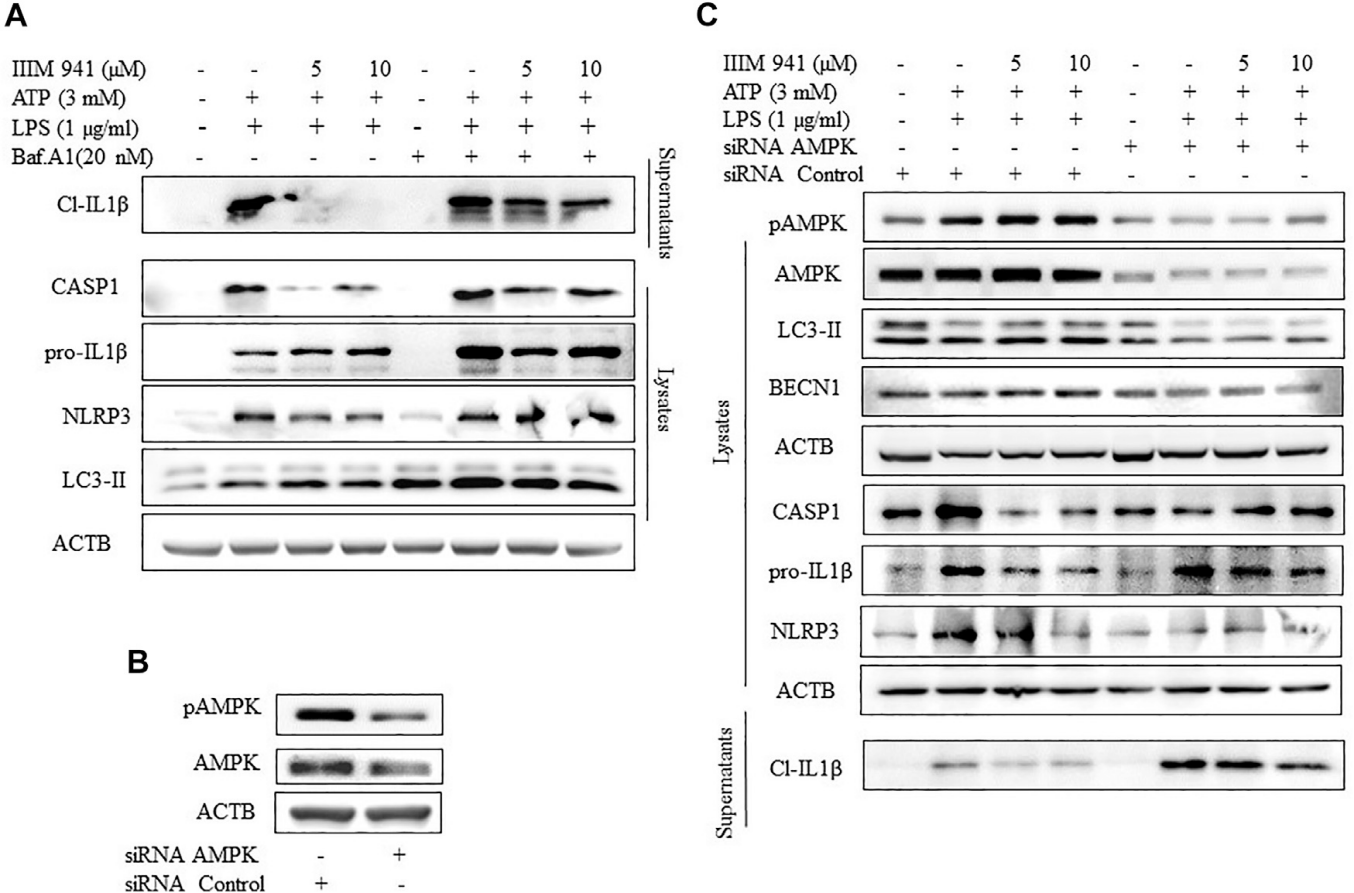
Effect of autophagy inhibition on IIIM-941 mediated inhibition of NLRP3 inflammasome **(A)** Western blot analysis of NLRP3 inflammasome proteins after inhibition of autophagy by a pharmacological inhibitor Baf A1 (20 nM), which was added 1 h prior to ATP stimulation of J774A.1 cells **(B)** Analysis of knock-down of AMPK after 24 h of siRNA transfection of J774A.1 cells **(C)** Effect of genetic knockdown of AMPK on IIIM-941 mediated induction of autophagy and inhibition of NLRP3 inflammasome in J774A.1 cells. The blots presented here are representative in nature. ImageJ software was used to quantify the band density of the western blot from three independent experiments and quantified data are presented in the Supplementary Figures S4A,B.

Further, to evaluate the relationship between IIIM-941 induced autophagy and the inhibition of NLRP3 inflammasome, we attempted to inhibit autophagy by using pharmacological inhibitor bafilomycin A1. The treatment of ATP activated J774A.1 cells with IIIM-941 for 1 h led to accumulation of LC3-II indicating inhibition of autophagy (Figure 5A). Further, bafilomycin A1 significantly reversed the inhibitory effect of IIIM-941 on cleavage and release of CASP1 and IL-1β, as observed in the supernatants (Figure 5A). Interestingly, there was an increased accumulation of NLRP3 and pro-IL-1β, which clearly implicated the involvement of autophagy in the inhibition of NLRP3 inflammasome caused by IIIM-941 (Figure 5A and Supplementary Figure S5A).

We further wanted to know about what happens to IIIM-941 caused inhibition of NLRP3 inflammasome if the autophagy is inhibited at initial stages. Therefore, we knocked down AMPK level by using siRNA in the J774A.1 cells (Figure 5B). We observed that the samples where the AMPK was knocked down displayed significantly reduced levels of IIIM-941 induced autophagy marker LC3-II and other proteins pAMPK and BECN1 involved in autophagy (Figure 5C and Supplementary Figure S5B). Thus, indicating reduced autophagy levels.

Further, the knockdown of AMPK had a clear reversal impact on the inhibition of NLRP3 inflammasome caused by IIIM-941, which was indicated by reduced release of cleaved IL-1β (Figure 5C and Supplementary Figure S5B) in the supernatants and reduced cleavage of CASP1 in the whole cell lysates.

### IIIM-941 Inhibited the activation of NLRP3 Inflammasome in ATP Mediated Peritoneal Inflammation, Uric Acid-Mediated Air Pouch Inflammation and MSU Foot Paw Oedema Models

**FIGURE 6:**
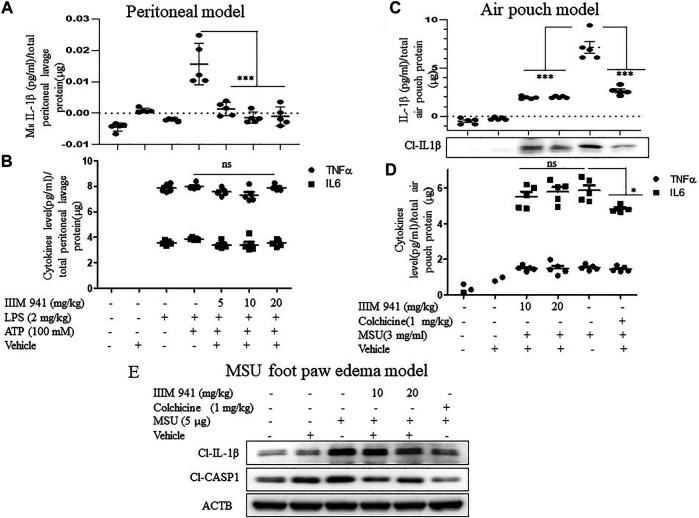
IIIM-941 displayed significant anti-NLRP3 inflammasome acitivity in different mice models **(A)** In the intraperitoneal inflammation model, ELISA measurement of IL-1β in the lavage collected from the intraperitoneal cavity of Balb/C mice (*n* = 5 in each group), which were intraperitoneally injected with IIIM-941 at the different doses of 5, 10, and 20 mg/kg and vehicle for 30 min prior to the LPS (2 mg/kg) treatment for 4 h and then stimulated with ATP (100 mM) for 15 min **(B)** Measurement of TNFα, and IL-6 in the peritoneal lavage **(C)** In the air pouch model of inflammation, **t**he Balb/C mice were injected with vehicle, colchicine (1 mg/kg), and 10, and 20 mg/kg of IIIM-941 for 30 min before the injection of MSU crystals (3 mg/ml) into the air pouches The release of cleaved IL-1β was measured in the fluid collected from air pouch by ELISA and western blotting **(D)** ELISA measurement of TNFα, and IL-6 in the air pouch lavage **(E)** Foot paw edema model in Balb/C mice was used to assess the oral efficacy of IIIM-941 at 10 and 20 mg/kg. Colchine (1 mg/kg p. o.) as a standard was also given before the injection of MSU (5 µg) in the right foot paw. The level of cleaved IL-1β and CASP1 were analyzed in the tissue taken from right foot paw. The quantification data of band density was analyzed by ImageJ software and is given in the Supplementary Figures S5A,B. Statistical analysis was done by using oneway ANOVA and post hoc Bonferroni test was applied. *p* value < 0.05 was considered significant with ***p < 0.001, **p < 0.01, *p < 0.05.

After confirming the mechanism of action of IIIM-941 *in vitro*, we validated the anti-NLRP3 activity of IIIM-941 in different *in vivo* models of NLRP3 inflammasome mediated inflammation. In the first model, the mice were given an intraperitoneal injection (i.p.) of IIIM-941 (5, 10, and 20 mg/kg) 30 min before the injection of LPS (2 mg/kg) for 4 h and then ATP (100 mM) for 15 min. The post-treatment ELISA analysis of peritoneal lavages revealed that NLRP3 inflammasome mediated release of IL-1β was strongly suppressed by the treatment with IIIM-941 at all the treatment doses (Figure 6A). However, IIIM-941 did not show any effect on LPS induced levels of TNF-α and IL-6 (Figure 6B). These data clearly proved that IIIM-941 inhibits NLRP3 mediated activation of IL-1β and it does not affect the levels of other pro-inflammatory mediators.

We used mouse air pouch model to examine the effect of IIIM-941 on MSU induced NLRP3 inflammasome. In this model, Balb/C mice were injected subcutaneously with sterile air to raise dorsum to make a pouch like structure. After seven days, different mice groups (*n* = 5) were injected into the air pouch with vehicle, colchicine (1 mg/kg), and the different doses of IIIM-941 (10, and 20 mg/kg) 30 min prior to injection of MSU crystals (3 mg/ml) for 6 h. The ELISA analysis of air pouch lavages clearly showed that MSU induced levels of IL-1β were highly suppressed in mice treated with IIIM-941 and the standard drug colchicine (Figure 6C). The western blot analysis of protein concentrated from lavage also show the similar results related to IL-1β release (Figure 6C and Supplementary Figure S6A). Whereas, the treatments did not affect the levels of either TNF-α or IL-6 (Figure 6D).

After confirming the anti-NLRP3 effect of IIIM-941 in two models where the drug was delivered *in situ*, we decided to analyze the oral efficacy of IIIM-941 in the MSU foot paw edema model of inflammation. The mice were treated with IIIM-941 at 10 and 20 mg/kg (p.o.) 30 min prior to the MSU injection in the foot pad of mice for 24 h. The Western blot analysis of foot paw tissue revealed significantly raised levels of cleaved IL-1β and CASP1, whereas, IIIM-941 at both the treatment doses significantly suppressed the cleavage of both IL-1β and CASP1 (Figure 6E and Supplementary Figure S6B), thus, clearly indicating the oral availability and efficacy of IIIM-941 against NLRP3 inflammasome. The standard drug colchicine also strongly abrogated the cleavage of IL-1β and CASP1.

## Discussion

We undertook this study to explore the anti-NLRP3 inflammasome activity of IIIM-941, which is a synthetic analog of an earlier reported estrogen receptor *β* (ER-β) agonist IIIM-983 (compound 11) (Mewshaw et al., 2005). ER-β signaling is reported to be involved in downregulation of inflammasome activity (Chakrabarti et al., 2014; de; Rivero Vaccari et al., 2016). Therefore, we synthesized a library of IIIM-983 analogs and screened all the compounds against NLRP3 inflammasome activity, data related to this was published recently (Abdullaha et al., 2021). IIIM-941 was identified as one of the most active compounds against NLRP3 inflammasome activity. Our initial data showed that IIIM-983 inhibits NLRP3 inflammasome only at the first step or before LPS priming, whereas IIIM-941 showed anti- NLRP3 inflammasome activity at both the steps that is before LPS priming and before NLRP3 activation by second stimuli. Further studies indicated that IIIM-941 does not affect the LPS induced levels of other pro-inflammatory cytokines including TNF-α and IL-6 in J774A.1 and BMDMs. NLRP3 inflammasome is involved in several chronic inflammatory conditions, where release of IL-1β causes sustained tissue damage (Lopez-Castejon and Brough, 2011; Lukens et al., 2012). However, assembly of NLRP3 inflammasome complex is an essential step for cleavage and activation of IL-1β (Martinon et al., 2009; He et al., 2016). Therefore, we looked into the effect of IIIM-941 on the proteins involved in NLRP3 inflammasome complex formation. We found that IIIM-941 was able to ablate the expression of NLRP3 and reduced its co-localization with CASP1 in J774A.1 cells. Further, oligomerization of ASC is a crucial step in the recruitment of pro-CASP1 (Coll et al., 2015; Dick et al., 2016). It was interesting to observe that IIIM-941 also reduced the oligomerization of ASC protein, thus preventing the cleavage and activation of CASP1.

Looking deep into understanding the mechanism of action, we asked if IIIM-941 can inhibit NLRP3 inflammasome by activation of autophagy. During the last few years significant number of studies have implicated autophagy as one of the crucial processes that regulate NLRP3 inflammasome activity in the cells (Dupont et al., 2011; Houtman et al., 2019). Autophagy can regulate NLRP3 inflammasome activity by several mechanisms, which includes autophagic clearance of mitochondria involved in generation of ROS (Zhou et al., 2011; Kim et al., 2014) and targeting components of NLRP3 complex such as NLRP3 and ASC (Heid et al., 2013; Wei et al., 2019). Besides, autophagy induction by rapamycin has also been reported to downregulate the expression of pro-IL-1β (Ko et al., 2017). We observed that IIIM-941 could activate autophagy in a time dependent manner as indicated by increased levels of LC3-II. Our data further indicated that induction of autophagy by IIIM-941 is mediated by activation of AMPK pathway, which is one of the primary regulators of autophagy. A significant inhibition of the downstream target of AMPK that is pMTOR (Ser 2448) was also observed in both time-dependent manner and under the conditions of NLRP3 inflammasome activation. It was interesting to note here that IIIM-941 did not activate AMPK by reducing the cell energy levels as indicated by increased ATP. We further found that IIIM-941 upergulated the AMPK pathway by calcium/calmodulin signaling, which was confirmed by the upregulation of pCAMKK2. It was interesting to observe in both J774A.1 and BMDMs that after the treatment with IIIM-941, increased expression of autophagic proteins was inversely related the expression of NLRP3 inflammasome proteins and release of IL-1β. After initial data about the involvement of autophagy in IIIM-941 mediated inhibition of NLRP3 inflammasome, we confirmed the role of autophagy by its pharmacological and genetic inhibition in NLRP3 regulation. J774A.1 cells treated with end stage autophagy inhibitor bafilomycin A1 displayed highly significant reversal of inhibitory effect of IIIM-941 on NLRP3 inflammasome. Additionally, the knock down of AMPK in J774A.1 cells led to downregulation of autophagy proteins including LC3-II and BECN1, while there was significant increase in the cleavage of CASP1 and release of IL-1β from the cells. Thus, implicating autophagy in the inhibition of NLRP3 inflammasome by IIIM-941. There are several neurodegenerative diseases such as Alzheimer’s disease (AD) and Parkinson’s disease (PD) where NLRP3 mediated chronic inflammation plays a crucial role in the pathological progression (Tan et al., 2013; Cho et al., 2014). Downregulation of autophagy has also been implicated in both AD and PD pathology, where it leads to reduced clearance of amyloid beta and alpha synuclein (Wani et al., 2019; Holbrook et al., 2021; Wani et al., 2021). Therefore, compounds like IIIM-941, which have the potential to target both inflammation and autophagy can raise new hope for the treatment of these diseases.

Further, after confirming the mechanism of action of IIIM-941, we attempted to validate its anti-NLRP3 inflammasome activity in different mice models. In the first model, IIIM-941 was able to significantly suppress the peritoneal release of IL-1β after intraperitoneal injection of ATP. Further, IIIM-941 also displayed strong inhibitory activity against MSU induced NLRP3 inflammasome in air pouch model. In both these models, IIIM-941 did not affect the raised levels of two important pro-inflammatory cytokines TNF-α and IL-6. Thus, further validating the inflammasome specific anti-inflammatory activity of IIIM-941. In both the above-mentioned models, treatment of IIIM-941 was given *in situ*, therefore, we wanted to know if IIIM-941 shows the same activity if given orally. To confirm this, we used MSU induced foot paw edema mice model, where the animals were given 10 and 20 mg/kg dose (p.o.) of IIIM-941. It was interesting to observe that IIIM-941 could inhibit the cleavage of CASP1 and IL-1β in paw tissue. Which implied that IIIM-941 could reach the inflamed paw tissue at effective concentration when given orally, thus, confirming the oral bio-availability of IIIM-941.

In conclusion, we identified IIIM-941 as a potential inhibitor of NLRP3 inflammasome, which displayed anti-NLRP3 inflammasome activity due to its ability to induce autophagy. Importance of autophagy and NLRP3 mediated inflammation in the pathology of neurogenerative diseases like AD and PD provides an opportunity to target these diseases with compounds like IIIM-941, which have ability to hit both these targets simultaneously. This approach may help in the development of new drugs against these diseases.

## Data Availability

The original contributions presented in the study are included in the article/Supplementary Material, further inquiries can be directed to the corresponding authors.

## Glossary

AMPK: AMP activated protein kinase
ASC: Apoptosis-associated speck-like protein containing a CARD
ATG: Autophagy related protein
ATP: Adenosine triphosphate
Baf A1: Bafilomycin A1
BECN1: Beclin1
BMDM: Bone Marrow Derived Macrophages
CAMKK2: Calcium/calmodulin-dependent protein kinase kinase 2
CASP1: caspase1
DAMPs: Damage-associated molecular patterns;
ECL: Enhanced Chemiluminescence
EDTA: Ethylenediamine tetraacetic acid
ELISA: Enzyme-linked immunosorbent assay
ER-β: Estrogen receptor-β
HEPES: 4-(2-hydroxyethyl)-1-piperazineethanesulfonic acid
HRP: Horseradish peroxidase
IL-1β: Interleukin-1β
IL-6: Interleukin-6
LC3: Microtubule-associated proteins 1A/1B light chain 3B
LPS: Lipopolysaccharide
M-CSF: Macrophage colony-stimulating factor
mt ROS: Mitochondrial Reactive oxygen species
mtDNA: Mitochondrial Deoxyribonucleic acid
MTT: 3-(4, 5, -dimethylthiazole-2-yl)-2, 5 diphenyltetrazolium bromide
NLRP3: NOD-like receptor protein 3
NP-40: Nonyl phenoxypolyethoxylethanol
PAMPs: Pathogen-associated molecular patterns
PEG: Polyethylene glycol
PMSF: Phenylmethylsulfonyl fluoride
PVDF: Polyvinylidene difluoride
RIPA: Radioimmunoprecipitation assay
SDS: sodium dodecyl sulfate
SDS-PAGE: Sodium dodecyl sulphate–polyacrylamide gel electrophoresis
siRNA: Small interfering RNA
TEMED: Tetramethylethylenediamine
TNF-α: Tumor Necrosis Factor alpha
