# Supplementary material for: IIIM-941, a Stilbene Derivative Inhibits NLRP3 Inflammasome Activation by Inducing Autophagy
Source: Front Pharmacol. 2021 Jun 25;12:695712. doi: 10.3389/fphar.2021.695712 (PMC8267097; doi:10.3389/fphar.2021.695712)
Supplement: Supplementary file 1 [file DataSheet1.docx]

**Supplementary Data**

**
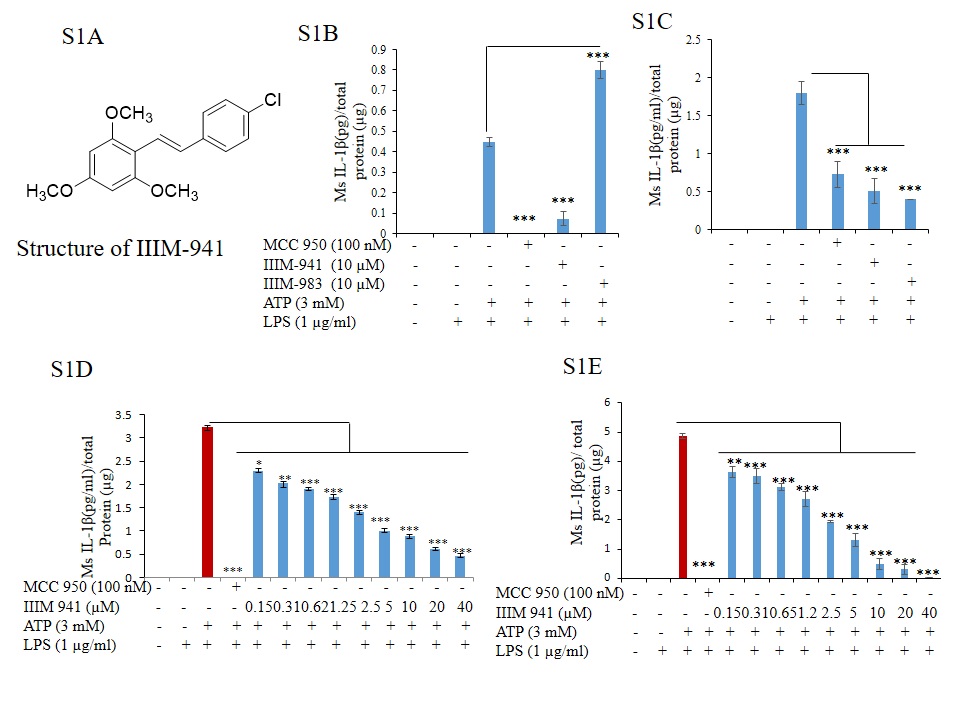
**

**Figure S1. (A)** Structure of IIIM-941. **(B)** Comparison of anti-NLRP3 inflammasome activity of IIIM-941 and IIIM-983 in J774A.1 cells, which were treated after LPS priming and before ATP stimulation of the cells and **(C)** when cells were treated before LPS priming. **(D)** Concentration dependent effect of IIIM-941 on NLRP3 inflammasome activity in J774A.1 cells and **(E)** in BMDMs. The release of IL-1β was taken as activation of NLRP3 inflammasome. **The data shown here are average ± SD of three independent experiments.** These data were used to calculate IC50 value of IIIM-941 against NLRP3.

Statistical significance was calculated by using one way ANOVA and Bonferroni test was applied as a post hoc. P value<0.05 was considered to be significant with ***p<0.001, **p<0.01, *p<0.05.


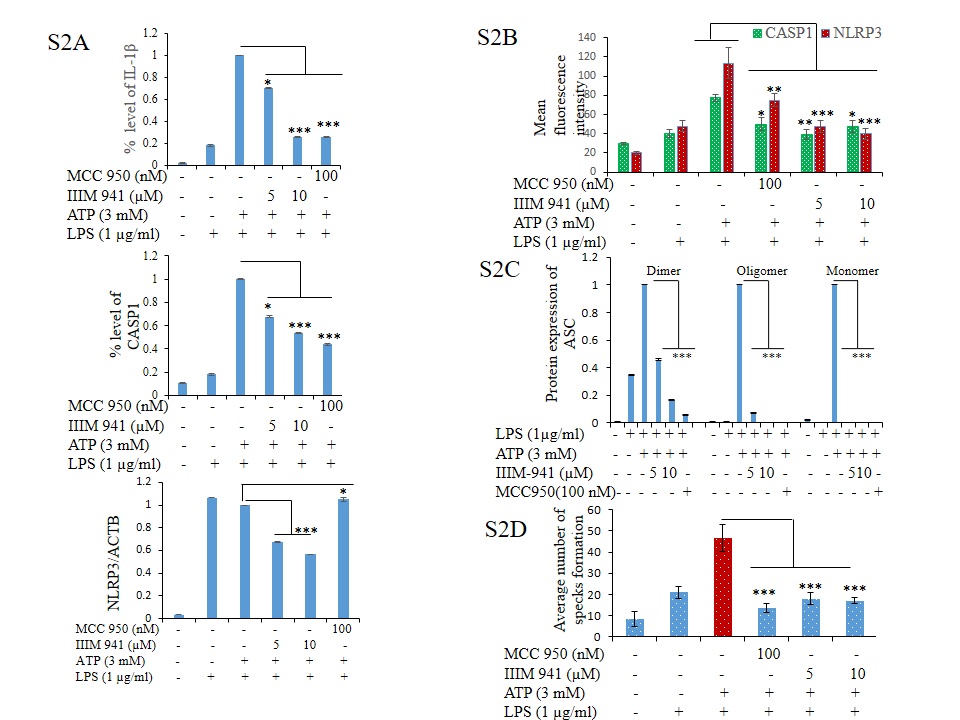


**Figure S2. (A)** Quantification of western blots from Figure 2A. **(B)** Quantification of mean fluorescence intensity of confocal images as given in Figure 2B. Fluorescent intensity was measured by using CellPathfinder software version 3.03.01.02. Green bars represent CASP1, and Red bars represent NLRP3. (C) Quantification of ASC oligomerization as observed in western blot image given in the Figure 2C. (D) Calculation of number of specks from the confocal images presented in Figure 2E. These data are an average of three independent experiments ± SD after various treatments as shown in the figure. Statistical significance was calculated by using one way ANOVA and Bonferroni test was applied to compare multiple samples. P value<0.05 was considered to be significant with ***p<0.001, **p<0.01, *p<0.05.


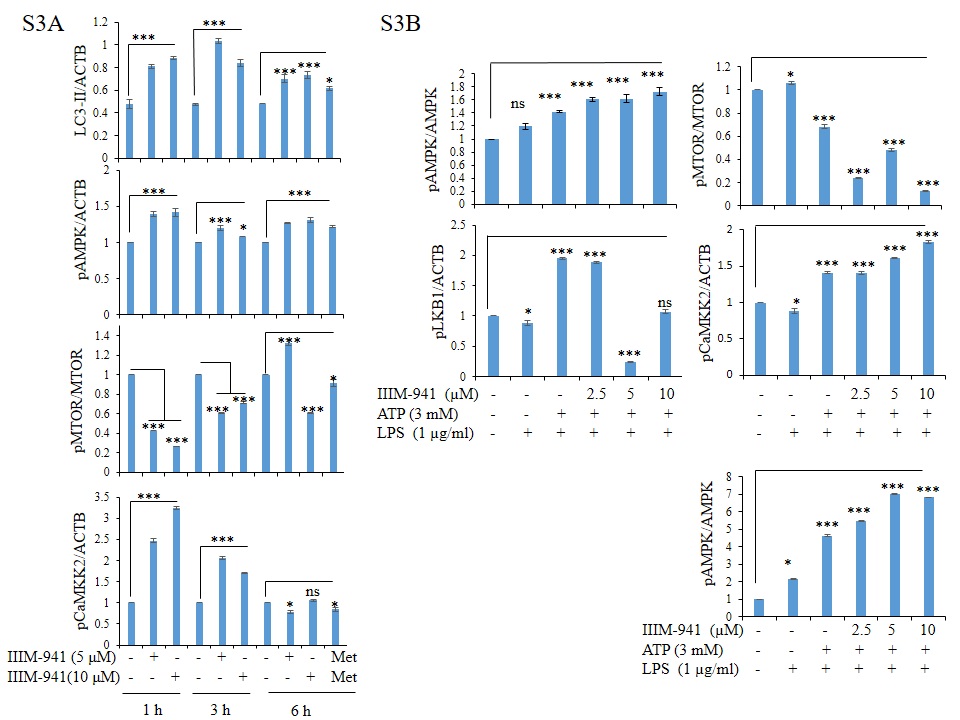


**Figure S3A and B. Quantification of western blots presented in the Figure 3.** ImageJ software was used to quantify the band density of western blots. The data presented here is the average ± SD of three independent experiments. Statistical comparisons were done by using one way ANOVA with Bonferroni test as a post hoc. P value<0.05 was considered significant. ***p<0.001, **p<0.01, *p<0.05.


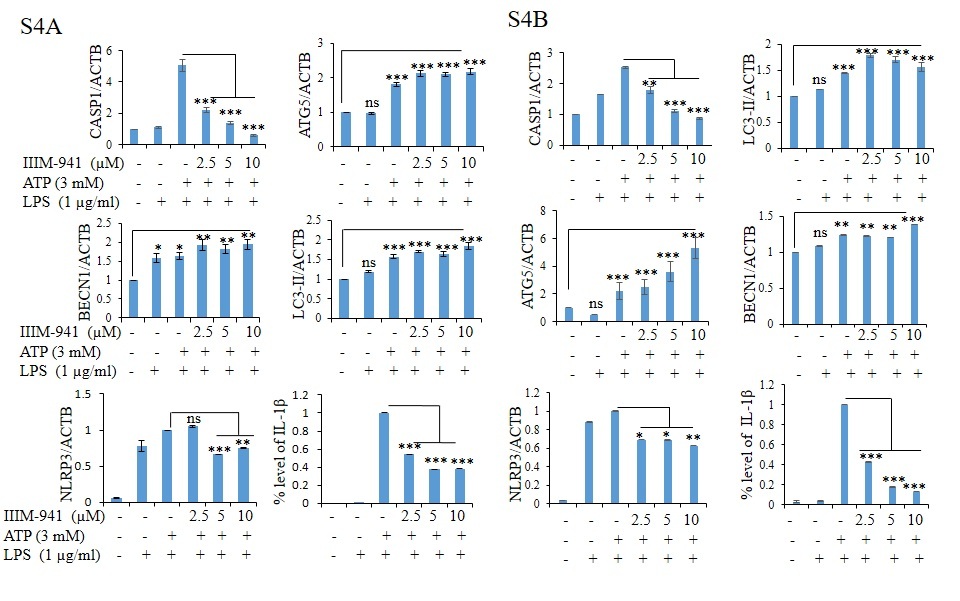


**Figure S4. Quantification of western blots presented in Figure 4. (S4A)** Western blot quantification was done by ImageJ software for the following proteins (LC3-II, ATG5, BECN1, NLRP3, CASP1, and cleaved-IL-1β) in J774A.1 cells and **(S4B)** LC3-II, ATG5, BECN1, NLRP3, CASP1 and cleaved-IL-1β in BMDMs. The expression of different proteins was normalized by dividing with the densitometry values of β-actin. The data presented here are an average of three independent experiments ± SD. Statistical comparisons were done by using one way ANOVA and Bonferroni test was applied as a post hoc. P value<0.05 was considered to be significant with ***p<0.001, **p<0.01, *p<0.05.


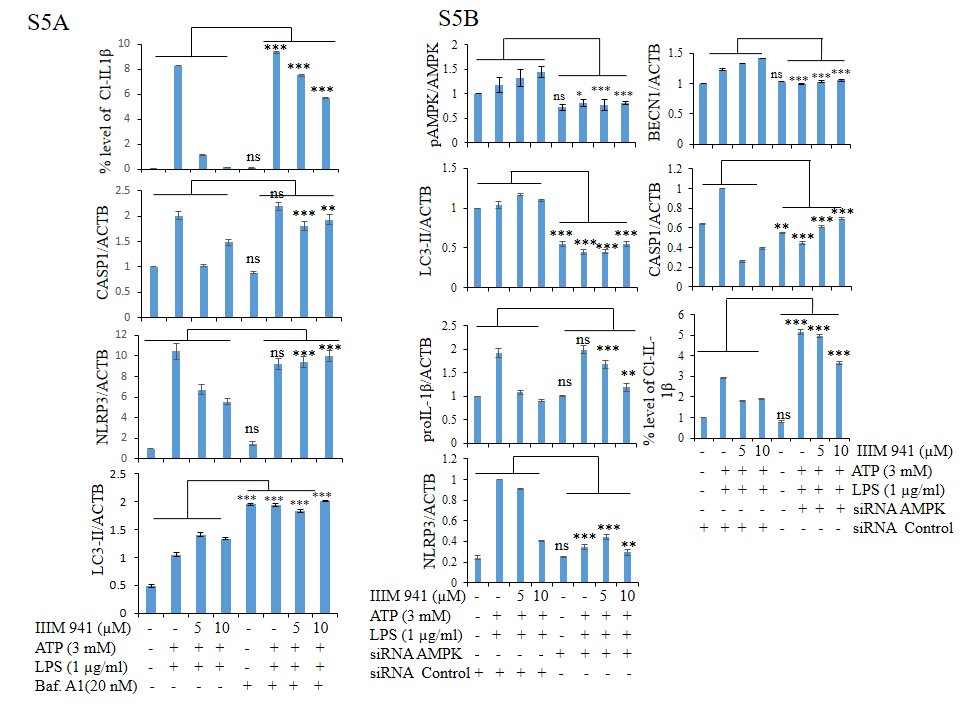


**Figure S5: Quantification of western blots presented in Figure 5 of the main manuscript. (S5A)** The expression of different proteins including LC3-II, NLRP3, CASP1, and Cl-IL-1β was quantified after treatment of J774A.1 with LPS and IIIM-941 (5 and 10 µM) treated in presence or absence of pharmacological inhibitor of autophagy Bafilomycin A1 (20 nM) 1 h before ATP stimulation. **(S5B)** The densitometry analysis of proteins pAMPK, BECN1, LC3-II, CASP1, pro-IL-1β, NLRP3, and cleaved IL-1β. The quantification was done by using ImageJ software. Protein expressions were normalized with the densitometry value of β-actin. The data shown here are an average of three independent experiments ± SD. Statistical comparisons were done by using one way ANOVA and Bonferroni test was applied as a post hoc. P value<0.05 was considered to be significant with ***p<0.001, **p<0.01, *p<0.05.


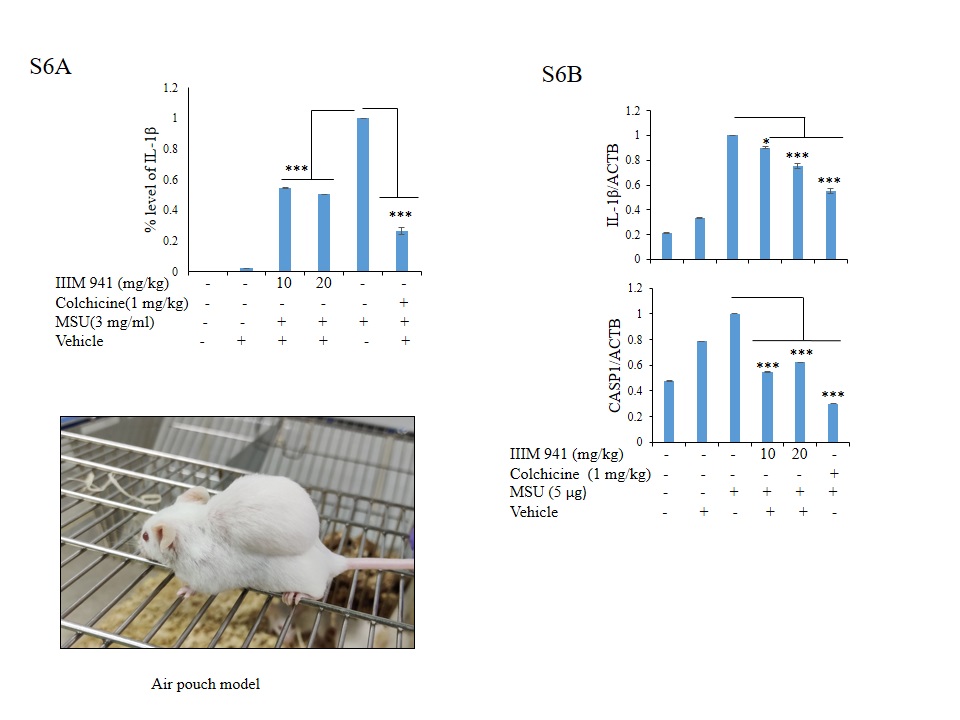


**Figure S6: (S6A)** Densitometry of western blot of cleaved IL-1β given in Figure 5C related to air pouch model, the data presented here is an average of three blots from different animals ± SD, and the image of one of the animals displaying raised air pouch. **(S6B)** The quantification of western blots of cleaved IL-1β and CASP1 form Figure 5E. The protein expression was analysed from the tissue taken form the foot paw of mice. Protein expressions were normalized with the densitometry value of β-actin. ImageJ software was used to quantify the band density of the western blots. Data presented here are and average of three independent experiments ± SD. Statistical comparisons were done by using one way ANOVA and Bonferroni test was applied as a post hoc. P value<0.05 was considered to be significant with ***p<0.001, **p<0.01, *p<0.05.
